# Identification of genes associated with cervical intervertebral disc degeneration and immune cell infiltration

**DOI:** 10.3389/fsurg.2026.1798328

**Published:** 2026-06-19

**Authors:** Dikai Bei, Kaifeng Gan, Binhui Chen, Jie Li

**Affiliations:** Orthopaedic Surgery, Ningbo Medical Center Lihuili Hospital Affiliated to Ningbo University, Ningbo, Zhejiang, China

**Keywords:** bioinformatics analysis, cervical intervertebral disc degeneration, diagnostic biomarkers, immune cell infiltration, machine learning

## Abstract

**Objective:**

This study aimed to identify novel diagnostic genetic biomarkers for early-stage cervical intervertebral disc degeneration (IDD) and to investigate the potential relationship between key genes and immune cell infiltration in IDD.

**Methods:**

mRNA expression profiles were obtained from the Gene Expression Omnibus (GEO) database. Differentially expressed genes (DEGs) between cervical IDD and control samples were identified using the linear model (limma R package). Functional annotation and pathway enrichment analyses were performed with Gene Ontology (GO) and Kyoto Encyclopedia of Genes and Genomes (KEGG). A Least Absolute Shrinkage and Selection Operator (LASSO) regression model and Support Vector Machine–Recursive Feature Elimination (SVM-RFE) were applied to screen potential biomarkers. Immune cell composition in IDD samples was estimated using the CIBERSORT method. Cervical disc specimens were collected from patients undergoing anterior cervical discectomy and fusion (ACDF) and classified into IDD and control groups based on MRI Pfirrmann grading. Quantitative PCR (qPCR) was performed to validate biomarker expression in these specimens.

**Results:**

A total of 71 DEGs were identified, including 50 upregulated and 21 downregulated genes in IDD samples. KEGG pathway analysis revealed significant enrichment in inflammation-related pathways. Through machine learning screening and experimental validation, we identified CDKN3, SLC22A4, and SYDE1 as key diagnostic biomarkers for IDD. Immune infiltration analysis suggested that these genes may contribute to IDD pathogenesis by modulating specific immune cell populations. qPCR results confirmed that CDKN3 expression was significantly downregulated in cervical IDD specimens (*P* < 0.05), whereas SLC22A4 and SYDE1 were significantly upregulated (*P* < 0.05).

**Conclusion:**

CDKN3, SLC22A4, and SYDE1 are associated with the pathogenesis and progression of cervical IDD, potentially through their regulatory effects on immune cell activity. These genes may serve as promising diagnostic biomarkers for cervical IDD and could aid in monitoring disease progression. Further studies are warranted to validate their clinical utility and elucidate the underlying mechanisms.

## Introduction

Neck pain is a significant global health burden, with a lifetime prevalence reaching 66% (1). Cervical intervertebral disc degeneration (IDD) is a major contributor to discogenic neck pain, accounting for 16%–41% of cases (2). The core pathological changes in IDD involve degradation of the extracellular matrix and excessive apoptosis of disc cells (3, 4).

In healthy intervertebral discs (IVDs), nerve fibers are confined to the outer annulus fibrosus (AF) (5). However, in degenerated discs, nerve fibers invade the inner AF and nucleus pulposus (NP)—a phenomenon known as neural invasion—likely driven by upregulated nerve growth factors during degeneration (6). These ingrowing fibers use substance P (SP) as a neurotransmitter, which has been closely linked to neck pain (7). Studies have shown a positive correlation between the density of blood vessels and nerves and the severity of disc degeneration (8). Whole-genome analyses indicate that expression of nerve-related genes increases alongside pro-inflammatory cytokine and chemokine-related genes in degenerated human AF (9). The NP, normally an avascular and immunoprivileged organ, becomes exposed to the immune system when degeneration disrupts the NP-blood barrier, leading to substantial immune cell infiltration (10).

Following IDD, intervertebral disc cells secrete elevated levels of inflammatory cytokines such as TNF-α, interleukins, and IFN-γ (11–14). These cytokines upregulate nerve growth factor (NGF) expression (15). For example, in the presence of IL-1β, NGF expression increases in human NP cells, and TNF-α upregulates SP expression (15). Elevated levels of NGF and SP are observed in painful human discs (16). These cytokines not only promote extracellular matrix degradation and chemokine production but also induce phenotypic changes in disc cells, amplifying the inflammatory cascade and contributing to discogenic pain (17). Immune cell infiltration has been implicated in the progression of IDD (18, 19), releasing additional inflammatory cytokines that further exacerbate the condition.

**Figure 1:**
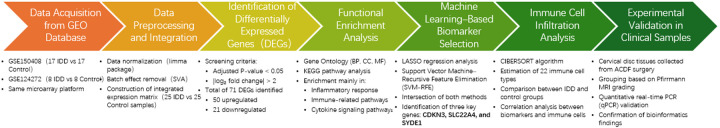
Methodological workflow for the identification and validation of diagnostic biomarkers in cervical intervertebral disc degeneration.

In this study, we aimed to identify diagnostic biomarkers for cervical IDD using bioinformatics analyses and to explore the relationship between key genes and immune cell infiltration. We analyzed GEO expression datasets to identify DEGs, performed GO and KEGG pathway analyses, and applied machine learning algorithms (LASSO and SVM-RFE) to screen potential biomarkers. Immune infiltration was assessed using CIBERSORT, and qPCR was used to validate biomarker expression in cervical IDD tissue specimens. The overall workflow of data acquisition, bioinformatics analysis, machine learning-based biomarker screening, immune infiltration analysis, and experimental validation is summarized in Figure 1.

## Materials and methods

### Gene expression data

mRNA expression profile data for cervical IDD were obtained from the GEO database (https://www.ncbi.nlm.nih.gov/geo/) for datasets GSE150408 and GSE124272. The GSE150408 dataset includes gene expression profiles for 17 IDD samples and 17 control samples, while GSE124272 includes 8 IDD samples and 8 control samples; both datasets were derived from the same microarray platform. All data preprocessing and analyses were performed in R (version 4.2.2) using Bioconductor packages (version 1.30.27). Data were formatted using Perl (Strawberry Perl 5.32.1.1) for compatibility with R. The R packages limma (20) and SVA (21) were employed to normalize the data and merge the two expression matrices, removing batch effects. The integrated gene expression matrix was then used for subsequent analysis of IDD vs. control samples.

### Identification of differentially expressed genes

Differentially expressed genes (DEGs) between cervical IDD and control samples were identified using the limma R package. The gene expression values were compared between the two groups, and statistical significance was assessed using a moderated *t*-test. Genes with an adjusted *P*-value < 0.05 and an absolute log2 fold change > 2 (|log2FC| > 2) were considered differentially expressed. Heatmaps and volcano plots of DEGs were generated using the pheatmap (version 1.0.13) and ggplot2 (version 4.0.3) R packages, respectively (22).

### Functional enrichment analysis

We performed functional enrichment analyses on the DEGs using the clusterProfiler R package (23, 24). Gene Ontology (GO) analysis encompassed Biological Process (BP), Cellular Component (CC), and Molecular Function (MF) categories. Kyoto Encyclopedia of Genes and Genomes (KEGG) pathway analysis was also conducted. Enriched terms and pathways with *P* <  0.05 were identified. GO and KEGG results were visualized using bubble plots.

### Biomarker identification via machine learning

To screen potential diagnostic biomarkers for cervical IDD, we employed two machine learning approaches. First, we applied the Least Absolute Shrinkage and Selection Operator (LASSO) regression model using the glmnet R package (25). This analysis selected a subset of DEGs with the strongest predictive power for distinguishing IDD from control samples. Second, we used Support Vector Machine–Recursive Feature Elimination (SVM-RFE) to further refine the list of DEGs (26). The SVM-RFE algorithm iteratively removed the least important features to optimize classification performance. We then identified overlapping genes selected by both LASSO and SVM-RFE as candidate biomarkers for IDD.

### Immune infiltration analysis

The CIBERSORT algorithm (https://cibersort.stanford.edu/) was used to estimate the relative proportions of 22 immune cell types in each sample (27). We compared the immune cell composition between IDD and control samples to identify significantly different immune cell populations. Associations between the expression levels of identified biomarkers and immune cell fractions were evaluated using Spearman correlation analysis.

### Cervical disc specimens and qPCR validation

**Figure 2:**
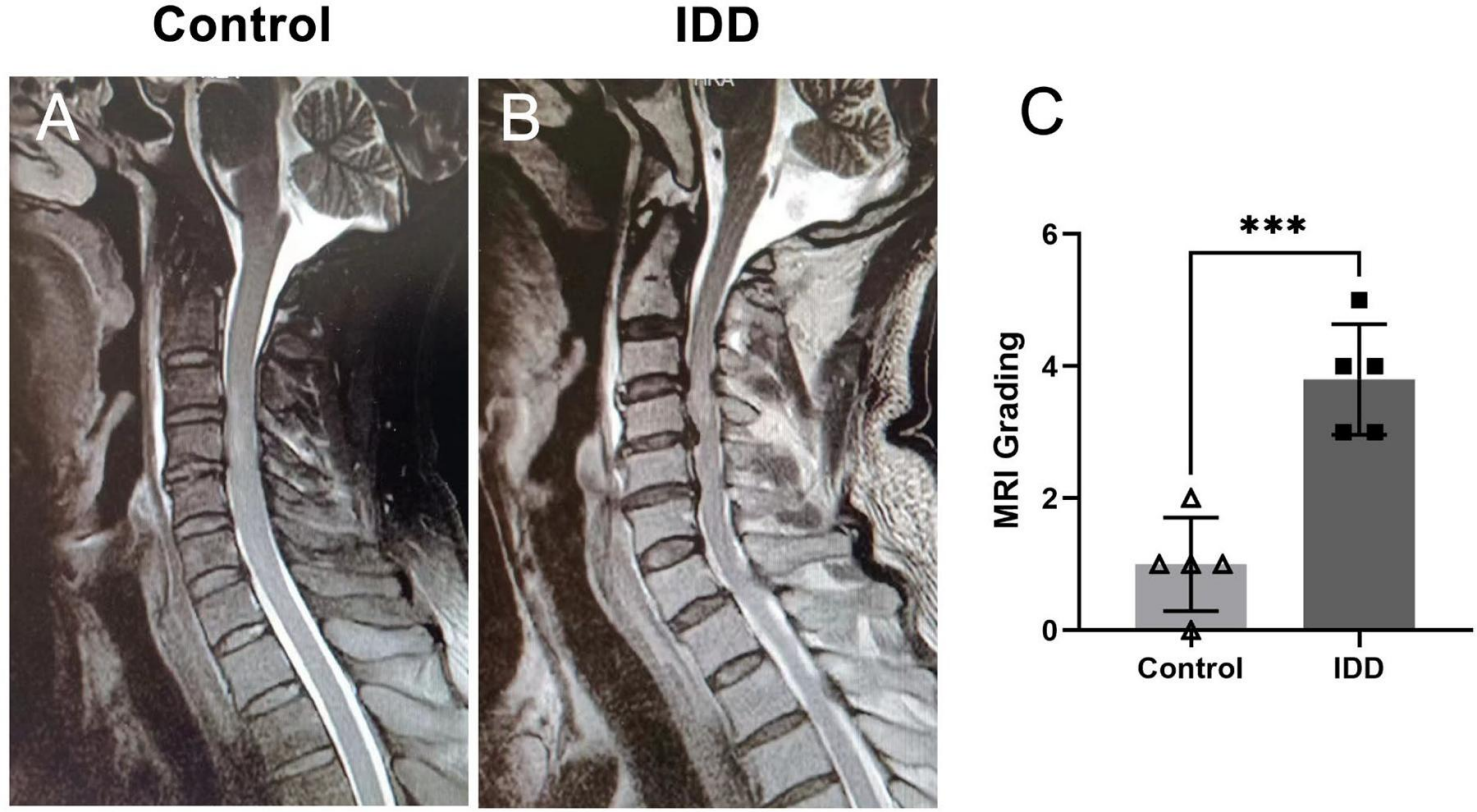
Cervical vertebra MRI images **(A)** for controlled group, **(B)** for IDD group. **(C)** MRI staging for intervertebral disc.

Cervical disc specimens were obtained from patients undergoing ACDF surgery. The IDD group (*n* = 5; 2 females, 3 males; aged 35–60 years) included patients with MRI-documented disc degeneration (Pfirrmann grade III or IV), while the control group (*n* = 5; 1 female, 4 males; aged 25–55 years) consisted of patients with cervical trauma but minimal degeneration (Pfirrmann grade II or lower) (28). Representative MRI images and Pfirrmann grading for both groups are shown in Figure 2. Disc tissues were collected following the diagnostic criteria of the Chinese Orthopedics Association (Spinal Column Group). Written informed consent was obtained from all participants, and the study was approved by the Medical Ethics Committee of Ningbo Medical Center Lihuili Hospital (Approval No. KY2023ML072).

Total RNA was extracted from disc tissues using a standard protocol. Complementary DNA was synthesized using reverse transcription. Quantitative real-time PCR was performed using gene-specific primers (Table 1) and a standard qPCR mix. GAPDH served as the internal control. Each sample was run in triplicate. Gene expression levels were calculated using the 2^-ΔΔCT method.

**Table 1:** Gene-specific primers.

Human gene	Primer sequence (5’–3’)
GAPDH	Forward **GGAAGCTTGTCATCAATGGAAATC**
	Reverse **TGATGACCCTTTTGGCTCCC**
CDKN3	Forward **CACCAGAGGGGAACTGTCAA**
	Reverse **TCAGGAGTCCCTCCATCTGC**
SLC22A4	Forward **CACTGCTGAGCTCTACCCAA**
	Reverse **CTACCCATGACGATGTAGGGC**
SYDE1	Forward **CAGCGGTCTGCCTATCTGAG**
	Reverse **ACCTTATACAGGGGCTGGGT**

### Statistical analysis

All statistical analyses were performed in R (version 4.2.2) and GraphPad Prism 9 (GraphPad Software, USA). Continuous variables were expressed as mean ± standard deviation. Differences between two groups were assessed using Student's *t*-test for normally distributed data or the Wilcoxon rank-sum test for non-normal data. Receiver operating characteristic (ROC) curves were generated using the pROC package to evaluate the diagnostic performance of candidate biomarkers. Correlations were assessed by Spearman's rank test. A *P*-value < 0.05 was considered statistically significant.

## Results

### Identification of differentially expressed genes

**Figure 3:**
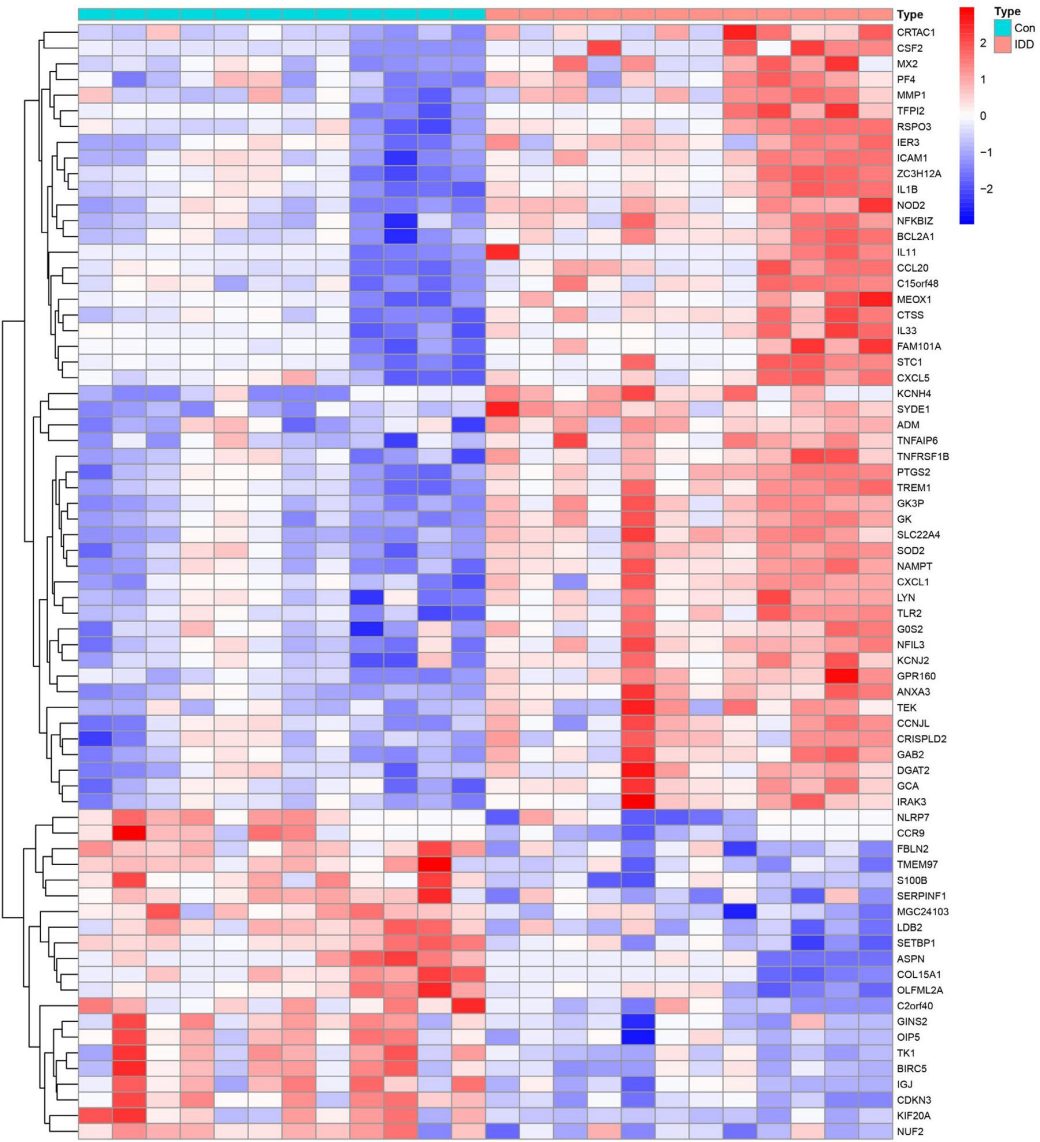
Heatmap of DEGs between cervical IDD and control samples. Red indicates upregulated genes; blue indicates downregulated genes. “Con” represents control samples; “IDD” represents cervical IDD samples.

**Figure 4:**
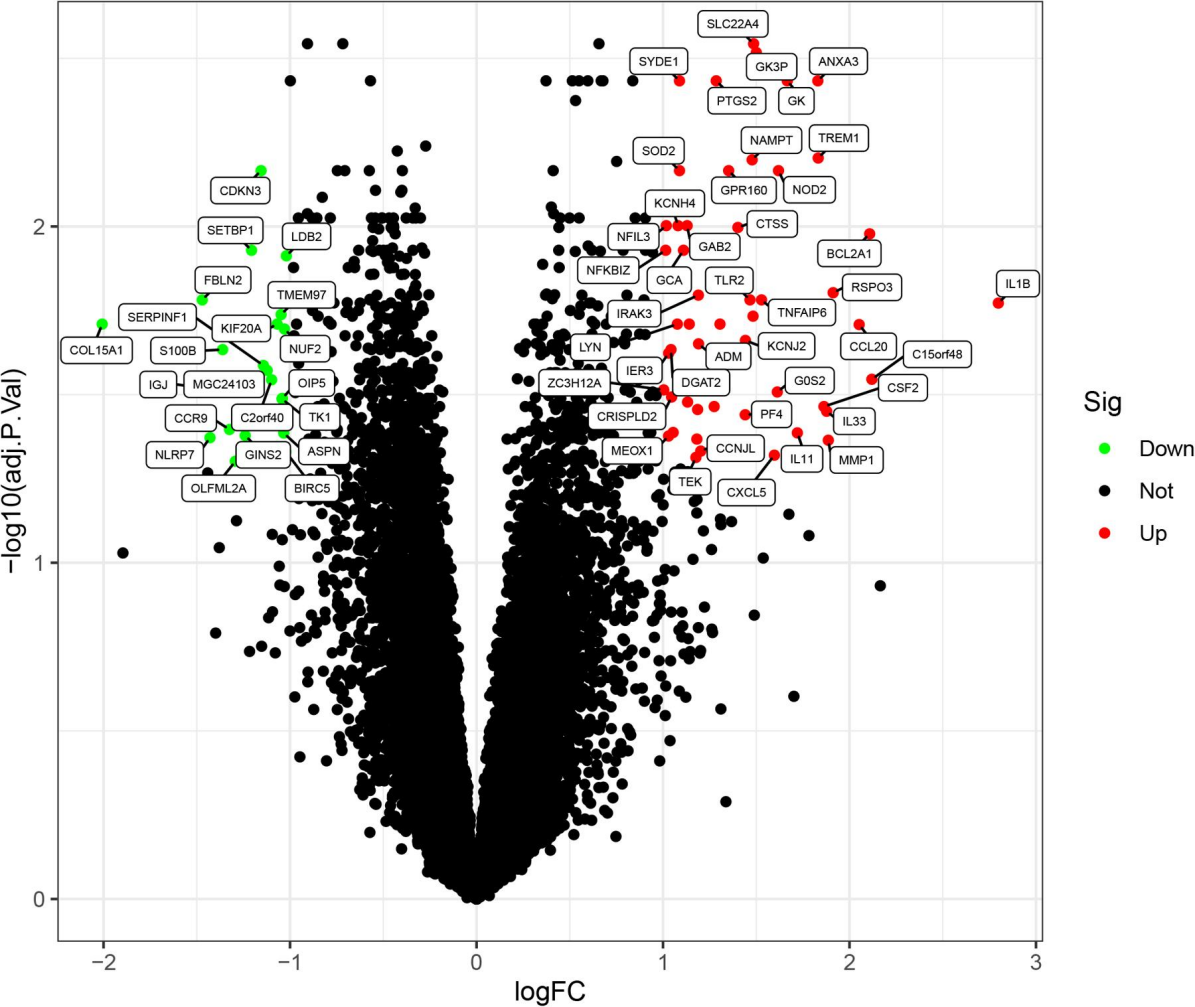
Volcano plot of DEGs. Red dots indicate significantly upregulated genes, and green dots indicate significantly downregulated genes in the IDD group.

After removing batch effects and integrating the data, we analyzed expression profiles from 25 cervical IDD samples and 25 control samples. A total of 71 DEGs were identified: 50 genes were significantly upregulated and 21 were downregulated in IDD samples compared to controls (Figure 3). The heatmap in Figure 3 and the volcano plot in Figure 4 illustrate the DEGs.

### Functional annotation and pathway enrichment

**Figure 5:**
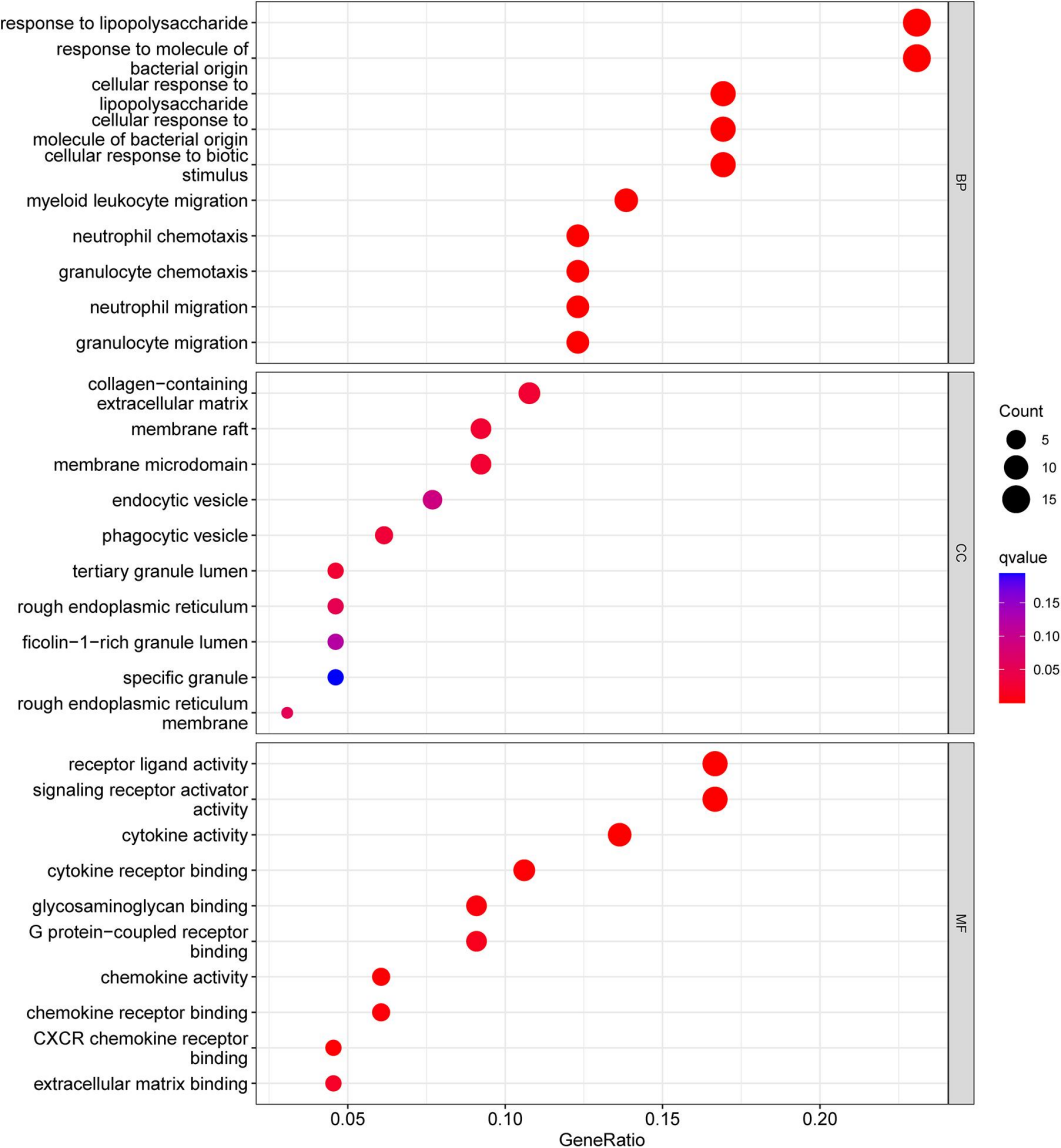
Bubble plot of GO functional enrichment analysis for the 71 DEGs. Bubble size represents the number of genes; color intensity represents enrichment significance.

**Figure 6:**
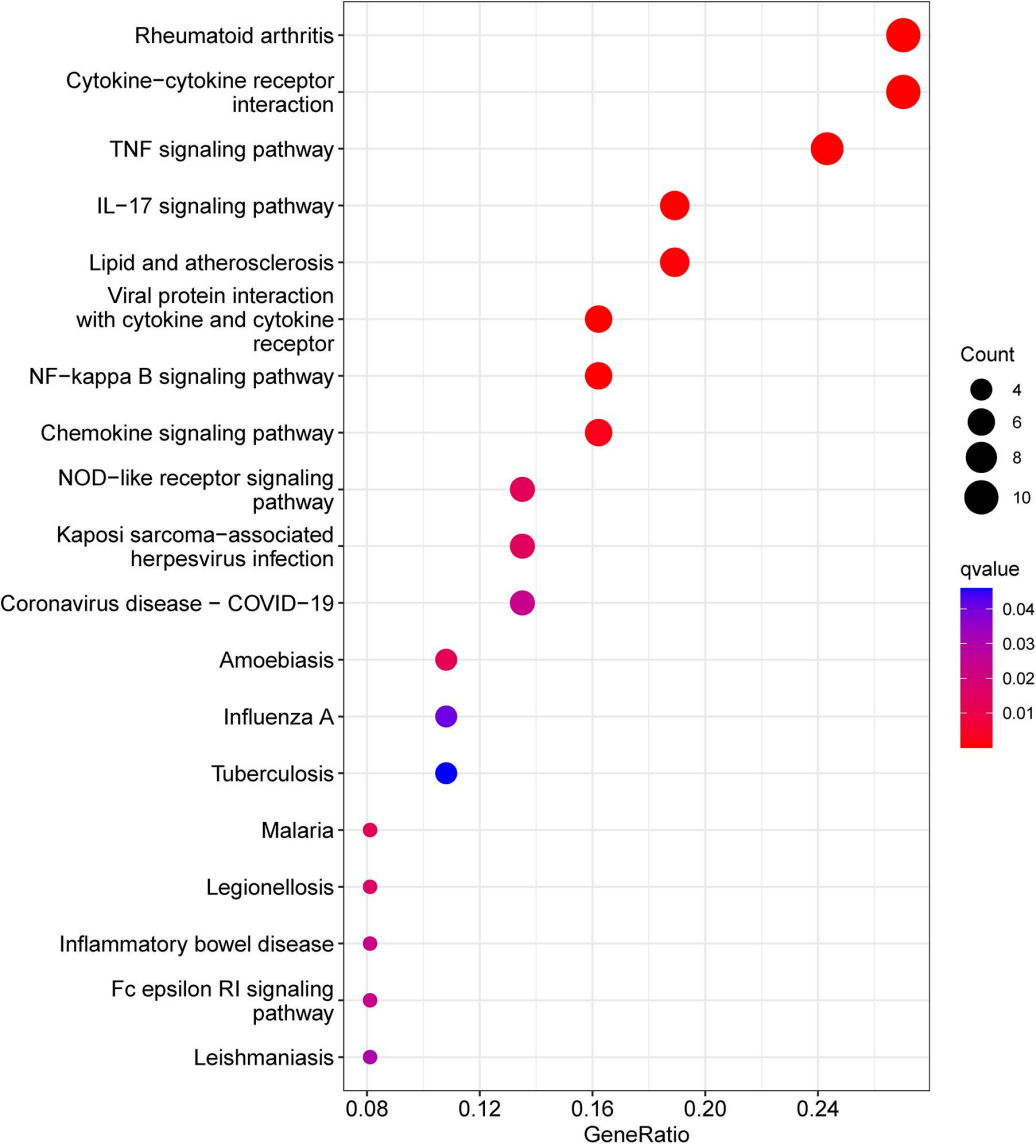
Bubble plot of KEGG pathway enrichment analysis for the 71 DEGs. Bubble size represents the number of genes; color intensity represents enrichment significance.

GO analysis revealed that the DEGs are primarily associated with immune and inflammatory processes. Enriched biological processes included responses to lipopolysaccharide (LPS), responses to bacterial molecules, neutrophil chemotaxis and migration, and leukocyte migration (Figure 5). Enriched molecular functions included extracellular matrix structural constituent and chemokine receptor binding. KEGG pathway analysis identified significant enrichment in inflammation-related pathways, such as rheumatoid arthritis, cytokine-cytokine receptor interaction, TNF signaling pathway, and IL-17 signaling pathway (Figure 6). These results suggest that the identified DEGs play critical roles in inflammatory and immune regulatory processes relevant to cervical IDD.

### Screening of diagnostic biomarkers

**Figure 7:**
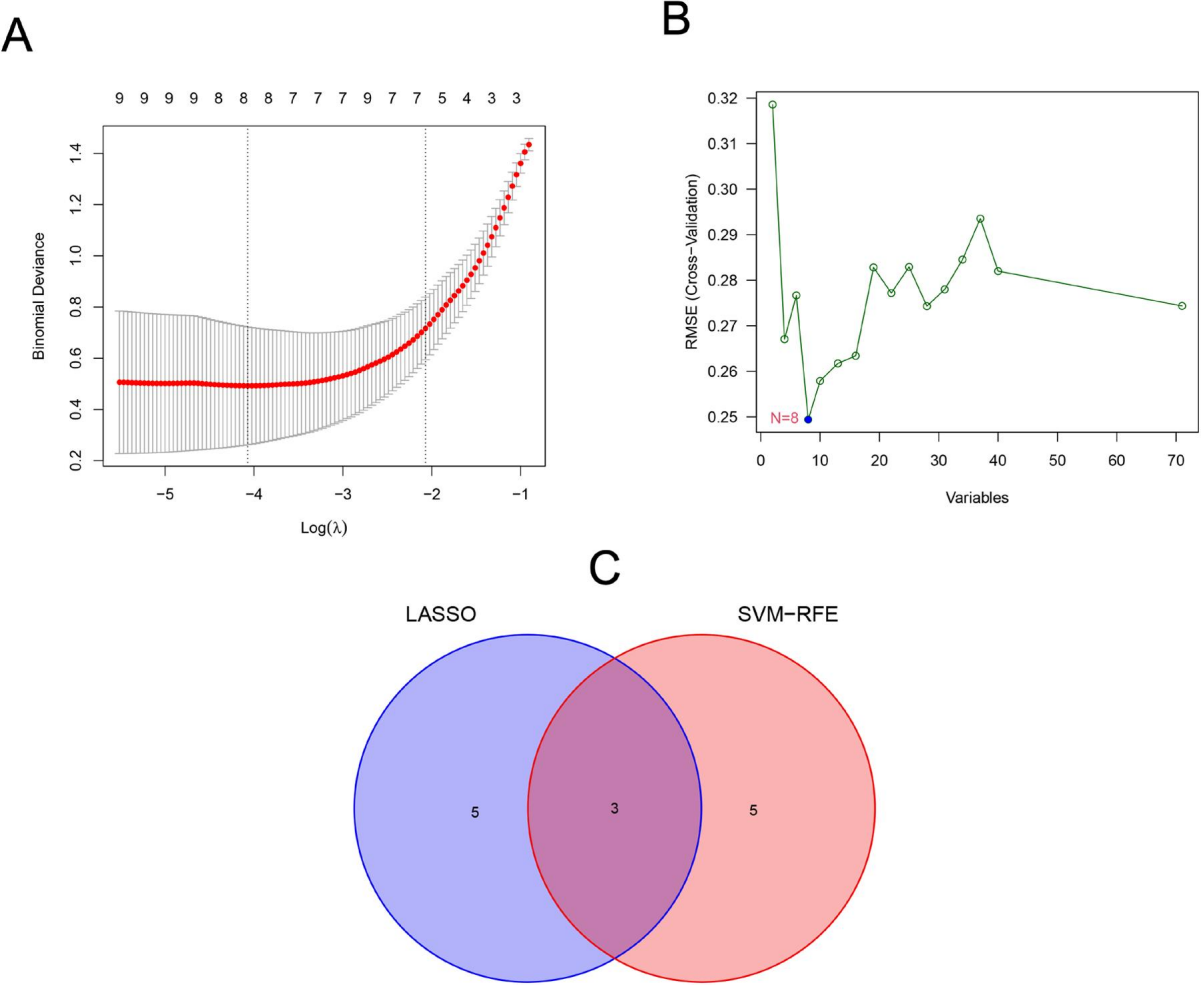
Selection of candidate diagnostic biomarkers for cervical IDD. **(A)** LASSO regression model identifying 8 potential biomarkers. **(B)** Feature selection by the SVM-RFE algorithm identifying 8 features. **(C)** Venn diagram showing the three genes (CDKN3, SLC22A4, SYDE1) identified by both methods.

To identify key biomarkers, we applied LASSO regression and SVM-RFE analyses. The LASSO model selected 8 candidate genes (Figure 7A), and SVM-RFE identified a subset of 8 features (Figure 7B). Integrating both methods, three overlapping genes were identified: CDKN3, SLC22A4, and SYDE1 (Figure 7C). These three genes were selected as candidate diagnostic biomarkers for cervical IDD. Their selection by two independent methods suggests they may play significant roles in IDD pathogenesis, although further functional validation is required.

### Expression and diagnostic value of biomarkers

**Figure 8:**
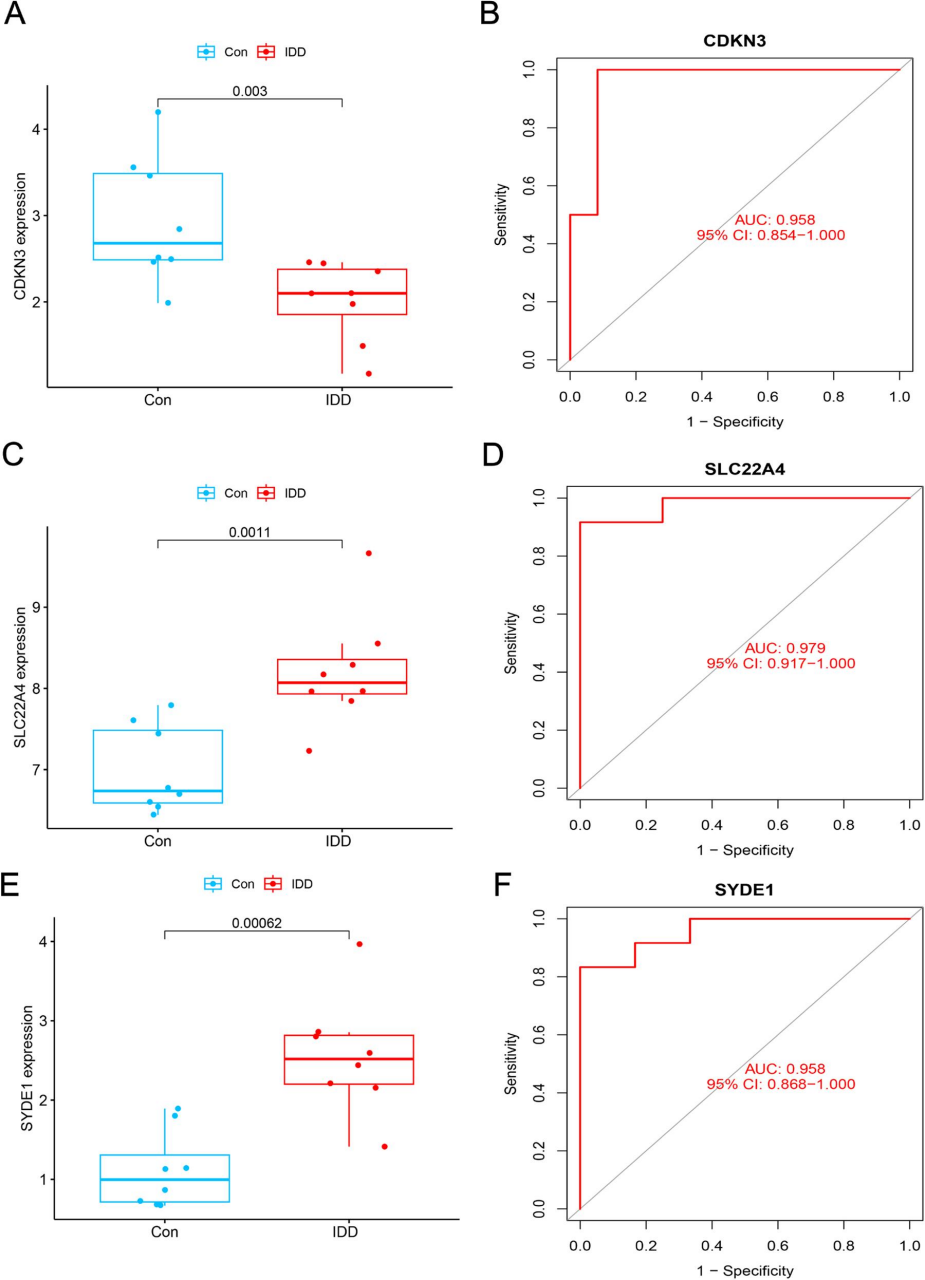
Expression and ROC analysis of biomarkers in cervical IDD. **(A)** CDKN3 expression is significantly lower in IDD samples than in controls. **(C,E)** SLC22A4 and SYDE1 expressions are significantly higher in IDD. **(B,D,F)** ROC curves for CDKN3, SLC22A4, and SYDE1, respectively. *P* < 0.05 indicates statistical significance.

We compared the expression levels of the candidate biomarkers between IDD and control samples. CDKN3 was significantly downregulated in IDD samples (Figure 8A), while SLC22A4 and SYDE1 were significantly upregulated (Figures 8C,E). To assess their diagnostic potential, we performed ROC analysis for each gene. The area under the curve (AUC) values were high for all three genes: 0.958 for CDKN3 (Figure 8B), 0.979 for SLC22A4 (Figure 8D), and 0.958 for SYDE1 (Figure 8F). These findings indicate that CDKN3, SLC22A4, and SYDE1 may serve as promising biomarkers for distinguishing cervical IDD from controls, although validation in larger cohorts is necessary.

### Immune infiltration analysis

**Figure 9:**
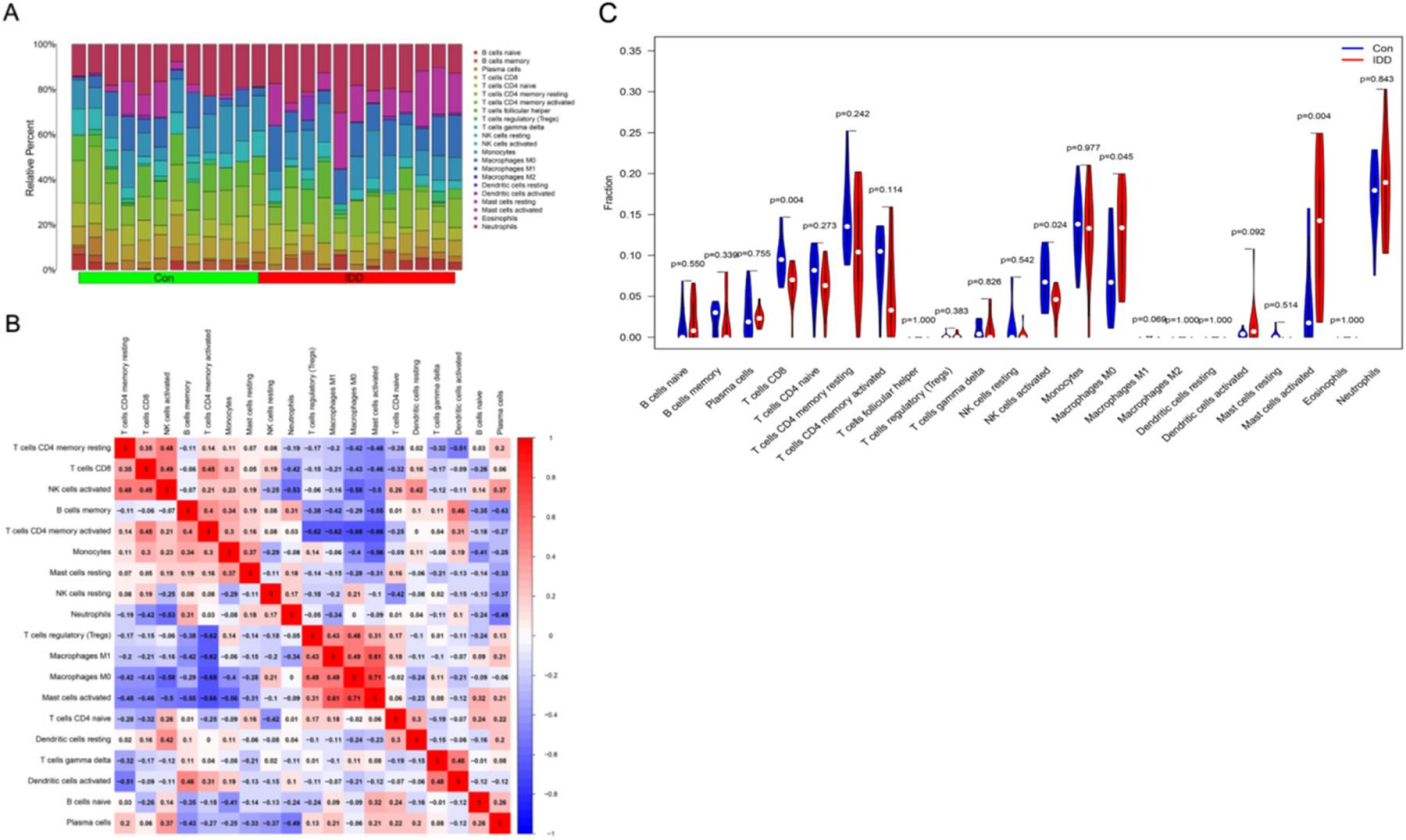
Immune infiltration analysis by CIBERSORT. **(A)** Proportions of 22 immune cell types in each sample (controls vs. IDD). **(B)** Correlation matrix of immune cell fractions. **(C)** Boxplots comparing key immune cell fractions between groups (*P* < 0.05 indicates significance).

Using the CIBERSORT algorithm, we analyzed the composition of 22 immune cell types in cervical IDD and control samples. The overall immune cell profiles for each sample are shown in Figure 9A, and the correlation between immune cell subsets is shown in Figure 9B. Notably, significant differences were observed in several immune cell populations: the IDD group had a higher proportion of M0 (unpolarized) macrophages and activated mast cells, whereas the proportions of CD8+ T cells and activated NK cells were lower compared to controls (Figure 9C; *P* = 0.045 for M0 macrophages, *P* = 0.004 for mast cell activation; *P* = 0.004 for CD8T cells, *P* = 0.024 for activated NK cells).

**Figure 10:**
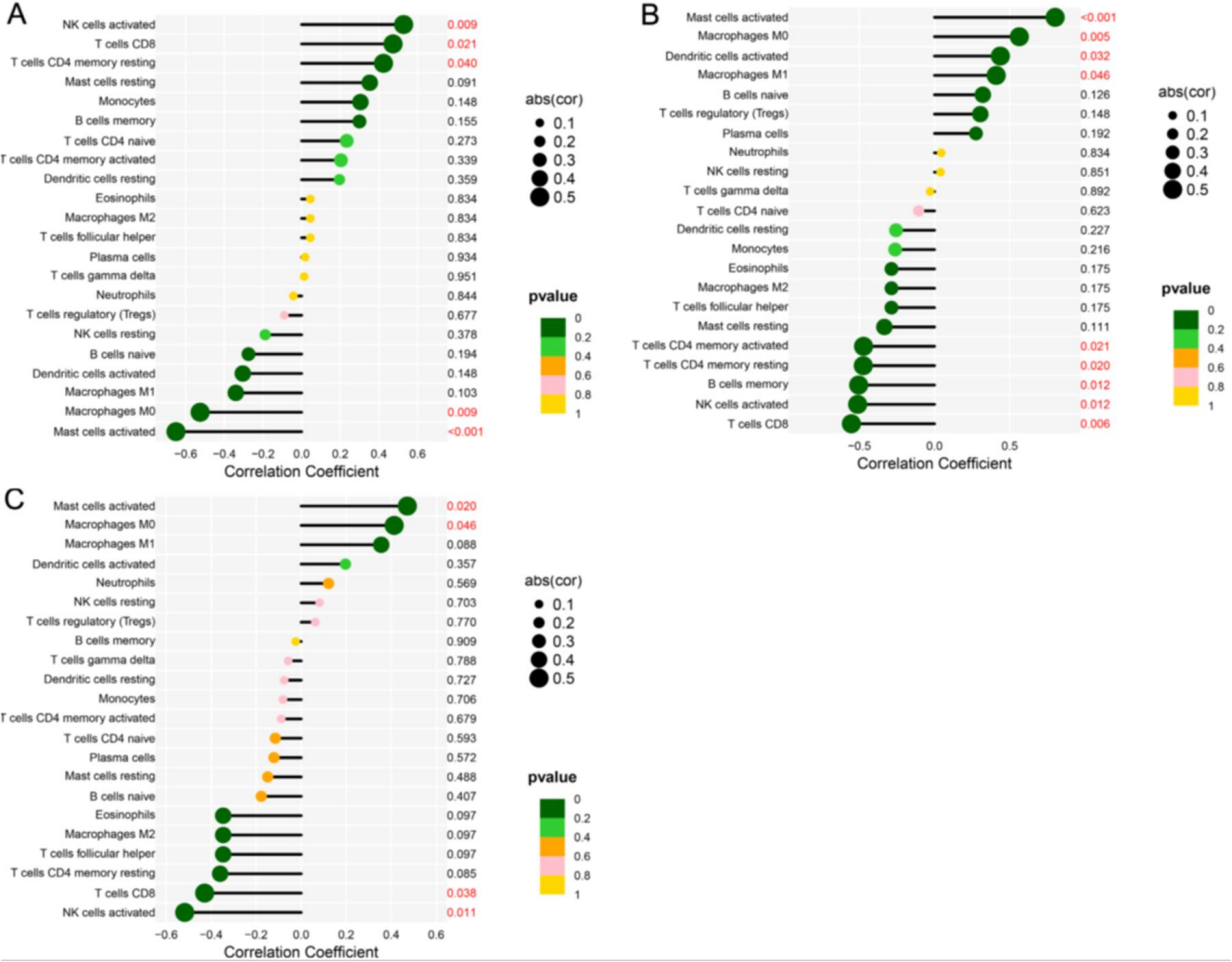
Correlation between biomarkers and immune cell fractions. **(A)** CDKN3, **(B)** SLC22A4, and **(C)** SYDE1 expression levels (horizontal axis) vs. immune cell proportions (vertical axis). Red indicates statistically significant correlations.

Next, we evaluated correlations between the expression levels of CDKN3, SLC22A4, and SYDE1 and the fractions of various immune cells. CDKN3 expression was positively correlated with the fractions of activated NK cells and resting CD4+ memory T cells, and negatively correlated with activated M0 macrophages and activated mast cells (Figure 10A; Table 2). SLC22A4 expression showed positive correlations with activated mast cells, M0 and M1 macrophages, dendritic cells, and CD4+ memory T cells, and negative correlations with CD8+ T cells, resting and activated CD4+ memory T cells, memory B cells, and activated NK cells (Figure 10B; Table 2). SYDE1 expression was positively correlated with activated mast cells, M0 macrophages, and CD8+ T cells, and negatively correlated with activated NK cells (Figure 10C; Table 2). These findings suggest that CDKN3, SLC22A4, and SYDE1 may influence the pathogenesis and progression of cervical IDD by modulating specific immune cell populations. However, further mechanistic studies are needed to fully elucidate these interactions.

**Table 2:** Detailed correlation coefficients and statistical values.

Gene	Cell	Cor	*P* value
CDKN3	T cells CD8	0.473	0.021*
	T cells CD4 memory resting	0.423	0.040*
	NK cell activated	0.528	0.009**
	Macrophages M0	−0.525	0.009**
	Mast cells activated	−0.650	0.001**
SLC22A4	B cells memory	−0.505	0.012*
	T cells CD8	−0.553	0.006**
	T cells CD4 memory resting	−0.474	0.020*
	T cells CD4 memory activated	−0.472	0.021*
	NK cells activated	−0.511	0.012*
	Macrophages M0	0.564	0.005**
	Macrophages M1	0.410	0.046*
	Dendritic cells activated	0.438	0.032*
	Mast cells activated	0.803	0.000***
SYDE1	T cells CD8	−0.429	0.038*
	NK cells activated	−0.517	0.011*
	Macrophages M0	0.413	0.046*
	Mast cells activated	0.471	0.020*

*P < 0.05, **P < 0.01, ***P < 0.005.

### qPCR validation of biomarkers

**Figure 11:**
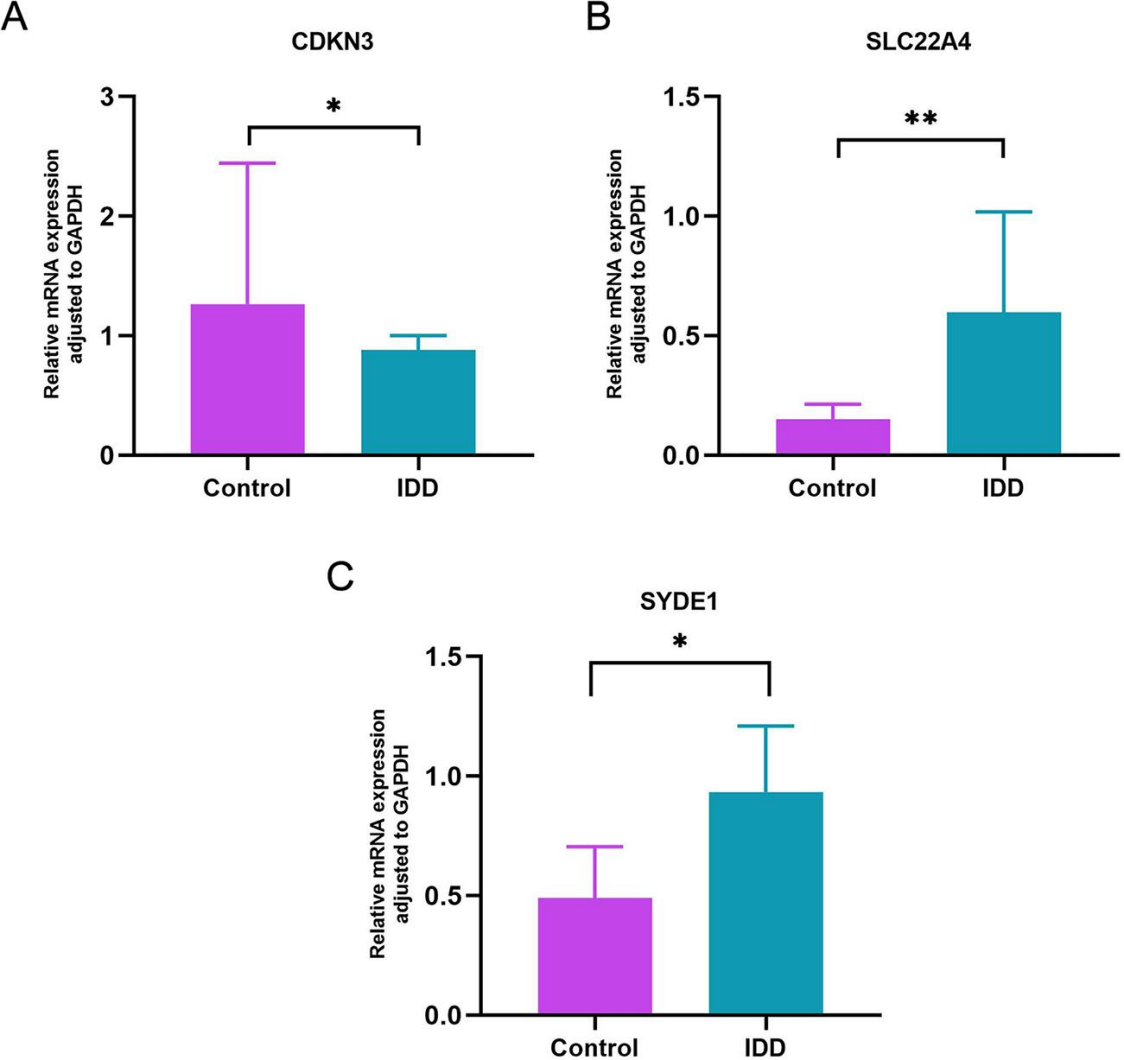
qPCR validation of biomarkers in cervical disc specimens. **(A)** CDKN3 expression (higher in control than IDD). **(B)** SLC22A4 expression (higher in IDD). **(C)** SYDE1 expression (higher in IDD). *P* < 0.05, *P < 0.01.

We performed qPCR on cervical disc specimens to validate the expression changes of the candidate biomarkers. Compared to controls, CDKN3 expression was significantly lower in IDD specimens (Figure 11A; *P* < 0.05). In contrast, SLC22A4 and SYDE1 were significantly upregulated in IDD specimens relative to controls (Figures 11B,C; *P* < 0.05). These results confirm the trends observed in the bioinformatics analysis and suggest that CDKN3, SLC22A4, and SYDE1 are involved in the pathogenesis of cervical IDD.

## Discussion

Cervical intervertebral disc degeneration (IDD) is a leading cause of neck pain and imposes a substantial health and economic burden (1, 2). IDD is characterized by an imbalance between catabolic and anabolic processes in the disc: pro-inflammatory cytokines degrade the extracellular matrix and alter disc cell phenotypes, leading to tissue degeneration and nerve sensitization (11–14). Chemokines released from degenerated discs facilitate immune cell infiltration and activation, exacerbating the inflammatory cascade. Early diagnosis and intervention in IDD are critical; however, current diagnosis often relies on MRI, which may lag behind early pathological changes (29). Hence, there is an urgent need for reliable biomarkers to enable early detection and guide therapeutic strategies.

In this study, analysis of GEO datasets identified 71 DEGs in cervical IDD, with GO and KEGG analyses highlighting their involvement in immune and inflammatory pathways. Using two machine learning approaches, we identified CDKN3, SLC22A4, and SYDE1 as potential diagnostic biomarkers. qPCR validation confirmed that CDKN3 is downregulated and SLC22A4 and SYDE1 are upregulated in cervical IDD tissues. CDKN3 belongs to the dual-specificity protein phosphatase family and is known to regulate cell cycle and proliferation (30). Its downregulation in IDD samples suggests a potential protective role of CDKN3 in maintaining disc cell homeostasis. SLC22A4 encodes an organic cation transporter; it has been linked to autoimmune inflammation in Crohn's disease (31) and is highly expressed in immune tissues (31, 32). The upregulation of SLC22A4 in IDD suggests that it may mediate pro-inflammatory processes contributing to degeneration. SYDE1 encodes a Rho GTPase-activating protein involved in cytoskeletal remodeling (33). Mechanical stress is a key factor in IDD, influencing cell responses via cytoskeletal changes (34). Thus, elevated SYDE1 in IDD may reflect altered mechanotransduction promoting degeneration.

Our immune infiltration analysis revealed significant differences between IDD and control discs. Specifically, activated M0 macrophages and mast cells were more abundant in IDD, while activated CD8+ T cells and NK cells were reduced. These findings are consistent with reports that CD68+ macrophages, neutrophils, and T cells infiltrate degenerated discs (35). The disc cells and infiltrating immune cells secrete neurotrophic factors such as NGF and BDNF, which sensitize dorsal root ganglion neurons and amplify pain signals via ion channels like ASIC3 and TRPV1 (36–38). In line with this, our analysis showed that CDKN3, SLC22A4, and SYDE1 expression correlates with specific immune cell populations. For example, CDKN3 was associated with activated NK cells and resting memory CD4+ T cells, whereas SLC22A4 correlated with multiple cell types including mast cells and macrophages. These correlations suggest that these genes may influence IDD progression by modulating the local immune response. Understanding these interactions could reveal new immunotherapeutic strategies for IDD.

Several limitations of this study warrant consideration. First, while we have identified significant correlations, the specific mechanistic links between the hub genes and immune cell infiltration remain to be functionally validated. As our current findings are primarily correlative rather than causative, further *in vitro* and *in vivo* investigations (e.g., gene knockdown or overexpression) are essential to elucidate these regulatory axes. Second, the clinical sample size for validation was relatively modest; thus, larger, multicenter prospective cohorts are required to further solidify the diagnostic utility and generalizability of these biomarkers.

Despite these constraints, our integrative bioinformatics and clinical validation approach provides a robust molecular and immunological foundation for understanding cervical IDD. More importantly, these results offer a conceptual framework for the next phase of our research. Specifically, we hypothesize that the SYDE1-mediated “mechano-immune” sensing axis plays a pivotal role in disc pathology, particularly given the unique multi-axial shear and rotational forces characteristic of the cervical spine. Furthermore, exploring the SLC22A4-driven metabolic-inflammatory bridge within the avascular disc environment, alongside CDKN3-related cellular senescence, will be critical. Collectively, these pathways represent our strategic transition from initial biomarker identification toward a comprehensive mechanistic elucidation of cervical disc degeneration.

## Conclusion

We identified three novel genes CDKN3, SLC22A4, and SYDE1 that are associated with cervical intervertebral disc degeneration and may serve as diagnostic biomarkers. These genes appear to influence IDD pathogenesis by modulating specific immune cell populations. The interplay between these genes and immune infiltration offers new perspectives on the molecular mechanisms underlying IDD and suggests potential targets for future immunotherapeutic interventions.

## Data Availability

The datasets presented in this study can be found in online repositories. The names of the repository/repositories and accession number(s) can be found in the article/Supplementary Material.
